# Occurrence of Antibiotic Resistance in *Lacticaseibacillus paracasei* and *Lactiplantibacillus plantarum* Strains Isolated from Traditional Sardinian Fermented Food

**DOI:** 10.3390/antibiotics15010018

**Published:** 2025-12-22

**Authors:** Gavino Carboni, Maria E. Mura, Margherita Chessa, Giuseppe Blaiotta, Anna Nudda, Nicoletta P. Mangia

**Affiliations:** 1 Department of Agriculture, University of Sassari, Viale Italia 39, 07100 Sassari, Italy; gcarboni1@uniss.it (G.C.); mariaelena.mura82@gmail.com (M.E.M.); marghe.chessa@tiscali.it (M.C.); anudda@uniss.it (A.N.); 2 Department of Agricultural Sciences, University of Naples Federico II, Via Università 100, 80055 Naples, Italy; blaiotta@unina.it

**Keywords:** meat and dairy products, *L. plantarum*, *L. paracasei*, antibiotics resistance, PCR analysis, plasmids

## Abstract

Background: This study investigated the phenotypic and genotypic antibiotic resistance profiles of 50 Lactic Acid Bacteria (LAB) strains—25 *Lactiplantibacillus plantarum* and 25 *Lacticaseibacillus paracasei*—isolated from traditional Sardinian fermented foods of animal origin. Methods: The sensitivity of the isolates to antibiotics such as β-lactams, tetracyclines, aminoglycosides, macrolides, phenicols, and glycopeptides was initially assessed using disc diffusion and minimum inhibitory concentration (MIC) tests. Subsequently, PCR analyses were performed on both genomic DNA and plasmid DNA to detect *blaZ*, *tet(W)*, *strA*, *aac(6′)-Ie–aph(2″)-Ia*, and *vanX* genes associated with resistance to ampicillin, tetracycline, streptomycin, gentamicin, and vancomycin. Results: The analysis revealed that *L*. *plantarum* strains frequently carried the *tet(W*) gene on the chromosome and *strA* on plasmids, while *vanX* was detected in most strains as a chromosomal determinant. By contrast, *L. paracasei* strains exhibited a predominantly plasmid-mediated distribution of resistance genes. For example, *strA*, *aac(6′)-Ie–aph(2″)-Ia* and *blaZ* were often found on plasmids, whereas *vanX* remained chromosomally encoded. Phenotypic assays confirmed high intrinsic resistance to vancomycin in both species, with *L. plantarum* showing a higher overall frequency and diversity of resistant phenotypes compared to *L. paracasei*. Conclusions: The co-occurrence of multiple resistance determinants, including plasmid-encoded ones, in most strains suggests that even autochthonous isolates from artisanal products may represent potential reservoirs for transmissible resistance genes.

## 1. Introduction

Antibiotics are among the most important therapeutic agents for the treatment of bacterial infections, having drastically reduced morbidity and mortality worldwide since their discovery [1]. However, the rapid emergence and dissemination of antibiotic resistance in human and veterinary medicine has become one of the most critical global health challenges [2,3]. Of particular concern is the spread of multidrug-resistant microorganisms that can evade multiple antibiotic classes used to treat them, as widely documented by several authoritative agencies [4,5]. For example, in Europe especially in Southern countries such as Italy, the latest ECDC report confirms persistently high resistance rates in major classes including fluoroquinolones, aminoglycosides and β-lactams [6].

Bacteria have evolved a variety of mechanisms to survive antibiotic pressure, including both intrinsic and acquired resistance. The relative predominance of these mechanisms depends on the type of antibiotic, its cellular target, and whether resistance determinants are chromosomal or carried by mobile genetic elements such as plasmids or transposons [7,8]. Horizontal gene transfer (HGT) plays a crucial role in this process, allowing resistance genes to move between taxonomically distant bacteria through conjugation, transformation, or transduction. The plasmid-mediated transfer of antibiotic resistance genes, in particular, has been identified as one of the main drivers of the global spread of antimicrobial resistance [9,10].

Although antibiotic resistance has historically been associated with pathogenic microorganisms, more attention is now being paid to non-pathogenic food-associated bacteria, including Lactic Acid Bacteria (LAB). These microorganisms, widely used in the production of fermented foods, are generally recognized as safe (GRAS) by the Food and Drug Administration (FDA) and hold Qualified Presumption of Safety (QPS) status according to the European Food Safety Authority (EFSA). However, they may act as reservoirs of resistance genes, some of which can be mobilized and potentially transferred to pathogenic or commensal bacteria within the food chain [11,12,13].

The potential threat to human health resulting from the inappropriate or excessive use of antibiotics in food-producing animals and in veterinary medicine is substantial [14], representing one of the most significant food safety risks associated with foods of animal origin.

Antibiotic consumption in cattle and poultry is increasing globally and, in developing countries, is feared to increase by 67% by 2030 [15].

In Europe in the decade from 2011 to 2021 there was a decline in the use of antibiotics in livestock by 47% (European Medicines Agency); evidently the legislative provisions aimed at regulating the use of antibiotics in veterinary medicine issued by the EU are proving effective. For instance, since 2006 the use of antibiotics as growth promoters has been prohibited (E.U. Regulation 1831/2003/EC). Recent EFSA, ECDC and EMA analyses indicate that reduced veterinary antibiotic use has been accompanied by declines in antimicrobial-resistant *Campylobacter*, *Salmonella* and commensal *E. coli* in several EU Member States, indicating a measurable impact on AMR trends [16]. Despite the implementation of these strategies, the surveillance on antibiotic resistance threat remains high, so much so that the same Authority strongly suggests further investigation of the non-pathogenic microorganisms as LAB involved in food fermentation.

Numerous fermented dairy and meat products rich in live lactobacilli could represent a vehicle for the entry of microorganisms into the human body. For instance, through the ingestion of fermented foods, lactobacilli can reach the gastrointestinal tract in numbers greater than 10^8^ CFU/mL [17], where they can interact with the resident microbiota and transfers genetic material, including genes of resistance to antibiotics [18].

Assessing the spread of antibiotic resistance in food-borne LAB is essential to verify the following: (1) the safety of using the microorganisms themselves; (2) the microbiological safety of the products; and (3) the potential risks throughout the entire food supply chain.

In this context, this study analyzed 50 isolates (25 *Lactiplantibacillus plantarum* and 25 *Lacticaseibacillus paracasei*) obtained from artisanal Sardinian sausages and cheeses, respectively, to investigate the safety, stability and potential transferability of their antibiotic-resistance determinants. These species were selected because they are predominant in these two traditional products and are commonly investigated for their potential use as starter cultures; therefore, assessing their safety is particularly relevant.

To achieve this, phenotypic and genotypic approaches were applied combining disc diffusion, MIC testing, and PCR-based gene detection on both chromosomal and plasmid DNA.

## 2. Results

In this work, phenotypic and genotypic antibiotic susceptibility of *Lactobacillus* strains isolated from animal-based food were analyzed. Overall, *Lactiplantibacillus plantarum* exhibited greater intra-species variability in antibiotic susceptibility, while *Lacticaseibacillus paracasei* showed more homogeneous resistance patterns, characterized by a predominance of plasmid-encoded determinants.

### 2.1. Phenotypic Antibiotic Susceptibility of L. plantarum Strains

The 25 *Lactiplantibacillus plantarum* strains isolated from traditional fermented meat exhibited heterogeneous antibiotic resistance profiles when tested by Agar-overlay Disc Diffusion (ADD) (Table 1). All isolates were resistant to vancomycin, as no inhibition zones were observed. In contrast, all strains were fully susceptible to chloramphenicol and erythromycin, showing inhibition halos greater than 18 mm (diameter), which served as quality controls. These results indicate the absence of phenotypic resistance to both antibiotics, and therefore no further MIC or genetic analyses were performed for these compounds.

In addition, variable susceptibility to β-lactam, aminoglycoside, and tetracycline antibiotics were revealed. Regarding ampicillin resistance (halo ≤ 12 mm), were displayed 3 isolates (12%) susceptible (S), 12 isolates (48%) moderately susceptible (MS), and 10 isolates (40%) resistant (R). Gentamicin resistance was detected in 14 isolates (56%), whereas the remaining 11 strains (44%) were fully susceptible by ADD. MIC testing revealed values exceeding the highest concentration (256 µg/mL). *L. plantarum* showed a mixed susceptibility profile for Streptomycin, with 8 isolates (32%) susceptible, 8 (32%) moderately susceptible, and 9 (36%) resistant. MIC values (64 µg/mL) were measurable only in four isolates, indicating MICs above the assay breakpoint. All *L. plantarum* isolates showed reduced susceptibility to tetracycline, with 18 out of 25 isolates (72%) classified as resistant (R) and 7 out of 25 (28%) as moderately susceptible (MS), and none fully susceptible (S). Only a few isolates exhibited measurable MIC values below the breakpoint (32 µg/mL), indicating susceptibility to tetracycline. For several strains, bacterial growth persisted at the highest antibiotic concentration tested, indicating MIC values > 256 µg/mL.

Overall, the comparison between ADD and MIC testing revealed method-dependent differences in the interpretation of susceptibility for several antibiotics. Notably, gentamicin showed a discrepancy of 44% between the two assays, with isolates classified as susceptible by ADD displayed MIC values that exceeded the highest concentration tested. A similar inconsistency was observed for streptomycin, for which eight isolates, corresponding to 32% of the dataset, classified as susceptible by ADD were instead resistant according to MIC testing. A further discrepancy was detected for tetracycline, where five isolates, representing 20% of the dataset, classified as resistant by ADD were instead susceptible according to MIC values. These findings highlight that the two phenotypic methods may not always provide concordant classification.

### 2.2. Phenotypic Antibiotic Susceptibility of L. paracasei Strains

The *Lacticaseibacillus paracasei* (*n* = 25) strains isolated from traditional dairy products exhibited a more uniform resistance profile than *L. plantarum* (Table 2). All isolates were resistant to vancomycin, gentamicin, and streptomycin, as confirmed by both ADD and MIC assays. *L. paracasei* isolates showed reduced susceptibility to ampicillin, with 22 out of 25 isolates (88%) classified as resistant (R) and 2 out of 25 (8%) as moderately susceptible (MS), and 1 out of 25 (4%) as fully susceptible (S). However, MIC testing, confirmed susceptibility for only a few isolates, highlighting a marked discrepancy between the two phenotypic methods. All isolates were susceptible to tetracycline (halo ≥ 19 mm, MIC 4 µg/mL), except for 9BC3, which showed a slightly reduced halo but still within the susceptible range. For *L. paracasei*, additional discrepancies between ADD and MIC testing were also observed. In the case of ampicillin, ten isolates, corresponding to 40% of the dataset, showed non-concordant classifications, with one strain classified as susceptible by ADD displaying an MIC value > 256 µg/mL and nine strains classified as resistant by ADD displaying MICs below the breakpoint threshold. Four isolates (16%) classified as susceptible by ADD showed MIC values exceeding the highest concentration tested for tetracycline, indicating resistance and further highlighting the lack of concordance between the two phenotypic methods.

### 2.3. Resistance Genes in Genomic and Plasmid DNA

The results of gene distribution patterns between chromosomal and plasmid DNA in both *L. plantarum* and *L. paracasei* strains are shown in Table 3 and Table 4.

Overall, *L. plantarum* exhibited heterogeneous antibiotic susceptibility and a mixed genetic architecture of resistance determinants, with both chromosomal and plasmid localization. The *tet(W)* gene, which was present in all 25 isolates, was located exclusively on the chromosome, which confirms that it is not mobile in these strains.

The presence of plasmid-associated *strA* in 20 strains is consistent with previous surveys reporting aminoglycoside resistance determinants in foodborne *Lactobacillus* spp., which are frequently located on plasmids or associated with mobile genetic elements [19]. The *aac(6′)-Ie–aph(2″)-Ia* gene was present in 13 strains, predominantly on plasmids (12/13) and only rarely on the chromosome (1/13). The *blaZ* gene, associated with β-lactam resistance, was detected in 6 isolates, all plasmid-borne. Finally, *vanX*, the glycopeptide resistance determinant, was found in 17 isolates, mostly on chromosomal DNA (16/17) and in a single case on a plasmid.

The predominance of *tet(W)* in the chromosome and the plasmid occurrence of *strA* and *blaZ* suggest a dual strategy of stable inheritance and potential horizontal gene transfer within this *L. plantarum* species.

A clear relationship was observed between the presence of specific genes and the corresponding phenotypic resistance, particularly for *strA*, *aac(6′)-Ie–aph(2″)-Ia* and *tet(W)*. Phenotypic-genotypic concordance for *vanX* displayed similarly good agreement, though not complete, and the only marked difference was observed for ampicillin. 

Genotypic analysis conducted on *L. paracasei* strains revealed the following distribution of resistance genes (Table 4).

The *tet(W)* gene was absent in all isolates, in agreement with the observed tetracycline susceptibility. The *strA* gene was detected in 24 isolates, located predominantly on plasmid DNA (18/24) and in a smaller number on the chromosome (6/24). The *aac(6′)-Ie–aph(2″)-Ia* gene was found in 14 isolates, all plasmid-borne, whereas *blaZ* was detected in 11 isolates, also exclusively plasmidial. Finally, the *vanX* gene, associated with glycopeptide resistance, was detected in 18 isolates, all located on chromosomal DNA.

*L. paracasei* strains displayed consistent intrinsic resistance to vancomycin and aminoglycosides as well as a plasmid-linked pattern of β-lactam resistance, while remaining largely susceptible to tetracycline. Approximately 63% of detected genes were found on plasmid, suggesting a higher potential for horizontal transfer than *L. plantarum*, although no mobile or conjugative elements were identified.

ADD phenotypic values were used to assess the agreement between phenotype and genotype. Concordance percentages were calculated by considering the ADD phenotypic data and PCR detection of the corresponding gene on the total number of strains per species (*n* = 25). 

**Figure 1:**
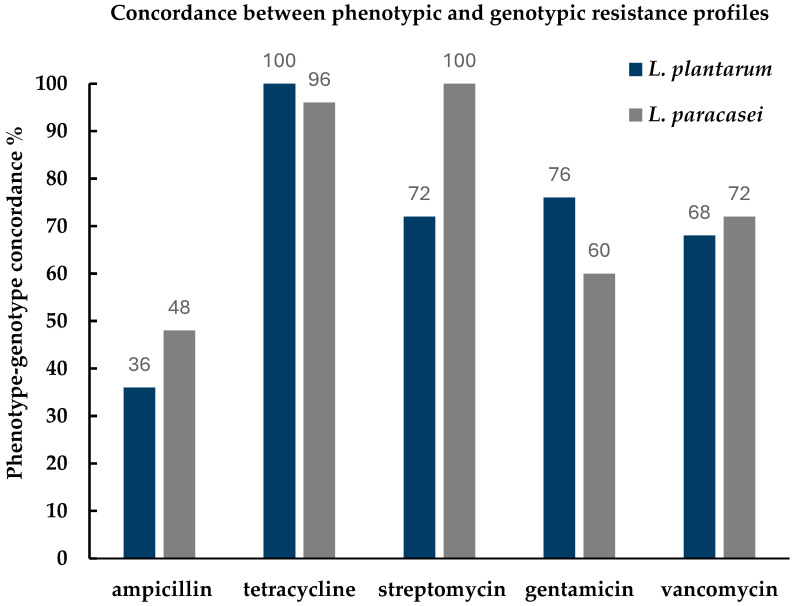
Phenotype–genotype concordance (%) for antibiotic resistance in *Lactiplantibacillus plantarum* and *Lacticaseibacillus paracasei*. Bars represent the percentage of isolates showing matching phenotypic (ADD) and genotypic (PCR) resistance profiles for each antibiotic tested (ampicillin, tetracycline, streptomycin, gentamicin, vancomycin).

The overall concordance between phenotype and genotype varied across resistance determinants. A high level of agreement was observed for streptomycin and tetracycline in *L. paracasei* whose presence strongly matched. For gentamycin and vancomycin, the concordance was slightly less consistent, with some mismatches emerging, ampicillin showed the lowest level of agreement in both *Lactobacillus* species (Figure 1).

## 3. Discussion

The food chain is considered one of the main reservoirs and transmission routes of antibiotic resistance genes (ARGs) [20,21,22]. Also, Lactic Acid Bacteria (LAB), which reach the gastrointestinal tract in high cell densities through the consumption of fermented products, may contribute to the spread or maintenance of resistant determinants within the human microbiota [23,24].

Furthermore, the physic-chemical conditions typical of fermented foods such as pH, salt concentrations, and technological stressors may modulate ARG expression and facilitate gene exchange among coexisting microorganisms [25,26]. Recent surveys on LAB from traditional foods indicate that resistance is not uncommon and often gene associated. As reported in the literature, 40.7% of LAB isolates from dairy products in Northern Italy harbored at least one antibiotic resistance gene, chiefly tetracycline determinants such as *tet(K)* and *tet(M)*, while intrinsic vancomycin non-susceptibility was nearly universal in *Lactobacillus* and *Leuconostoc* species [27].

In this study, *Lactiplantibacillus plantarum* and *Lacticaseibacillus paracasei* strains were isolated from specific traditional Sardinian fermented products. *L. plantarum* strains originated from spontaneously fermented sheep sausages, while *L. paracasei* strains were obtained from Casizolu, a traditional pasta-filata cheese produced from raw cow’s milk through natural lactic fermentation.

When comparing phenotypic results, minor discrepancies between ADD and MIC may occur because the two assays differ in their principles and interpretative criteria. ADD relies on antibiotic diffusion and halo diameter thresholds, whereas MIC determines inhibition based on a quantitative concentration gradient. As reported for LAB, medium composition, diffusion behavior and growth kinetics can influence halo formation and lead to borderline or non-overlapping classifications between methods [28]. In our dataset, ADD and MIC showed overall good agreement, with only occasional divergences, reflecting known methodological limitations in cross-method concordance.

Both lactobacilli species exhibited species-specific resistance patterns reflecting distinct resistance architectures, with a mixed chromosomal–plasmid distribution in *L. plantarum* and a predominantly plasmid-associated pattern in *L. paracasei*. At the same time, the detection of plasmid-associated genes indicates that these populations remain connected to the broader resistome and may retain a limited but non-negligible potential for gene mobilization under specific conditions [29].

During the initial PCR screening performed on total DNA extracted from colonies, several resistance genes showed weak or absent amplification despite the corresponding phenotypic resistance. Since total-DNA preparations may contain plasmid sequences at very low relative abundance, as reported in previous studies [30,31], plasmid DNA was subsequently extracted from all strains. This analysis was part of the planned workflow to determine the genomic location of the resistance determinants, and the use of plasmid-enriched templates allowed consistent detection of the target genes and their assignment to either the chromosomal or plasmid fraction.

Overall, the comparison between phenotypic and genotypic profiles revealed a generally good level of concordance although some mismatches were identified especially for ampicillin. Inconsistencies emerged, where resistant lactobacilli lacked detectable genes or susceptible strains carried them. Several explanations can be proposed: (i) the PCR screening targeted only a limited set of loci [32]; (ii) sequence variability in autochthonous isolates might have led to primer mismatches, causing false negatives [33]; (iii) some genes may be weakly expressed or silent under laboratory conditions, resulting in genotypic positivity without a corresponding phenotype [19]; and (iv) alternative intrinsic mechanisms, such as cell wall modifications, efflux pumps, or metabolic compensation, may confer resistance independently of the tested genes [34]; (v) PCR may also miss low-copy genes or plasmids present below the detection threshold, leading to false negatives [35].

These discrepancies between the genotypic analysis by PCR and the phenotype methods have already been reported in research conducted on LAB from poultry and swine meat products [36]. Similarly, Campedelli et al. [33] obtained different results between phenotypes and genotypes for vancomycin in *Lactobacilli* sp. The authors assume that as reported above could be due to the presence of alternative resistance mechanisms or to alteration of gene expression.

Most *Lactobacillus* species exhibit intrinsic resistance to aminoglycosides and vancomycin, as widely documented in the literature.

Overall, our results indicate that all lactobacilli strains were resistant to vancomycin, a well-established intrinsic trait of many LAB species. As widely documented in the literature, this resistance is associated with D-Ala–D-Lac–type modifications of the peptidoglycan terminus, which reduce vancomycin binding [37,38,39]. Because this intrinsic mechanism does not rely on the *vanX* locus, the presence of *vanX* is not predictive of phenotypic resistance, thereby explaining the observed phenotype–genotype discordance [34]. This trait is encoded chromosomally and is considered non-transferable according to EFSA guidelines [40]. Tetracycline resistance was also detected in several isolates of *L. plantarum* and none of *L. paracasei* showed resistance. The *tet(W)* gene was consistently identified at the chromosomal level in *L. plantarum*, confirming its non-transferable nature and supporting previous findings that reported *tet(W)* as one of the most common determinants in lactobacilli from fermented foods [34,41].

*L. plantarum* and *L. paracasei* strains each harbored multiple plasmid-associated resistance genes (e.g., *strA*, *aac(6′)-Ie–aph(2″)-Ia*, *blaZ*), although this trait was more pronounced in *L. paracasei.* Intrinsic resistance to aminoglycosides, such as gentamicin and streptomycin, is widespread among *Lactobacillus* species and is mainly attributable to reduced permeability of the cell envelope and low affinity of ribosomal targets for these antibiotics, rather than to the acquisition of specific resistance genes [33,41].

Although some resistance genes were located on plasmids, no experiments were performed to assess the presence of conjugative elements, transposons, or other mobility-associated features. Therefore, our data do not provide any experimental evidence for gene mobility, and no conclusions can be drawn regarding the horizontal transfer potential of these plasmids. However, evidence from Toomey et al. (2010) reported the transfer of *tet(M)* from *L. plantarum* isolated from a pork abattoir to *Lactococcus lactis* and *Enterococcus faecalis* strains can occur under optimized laboratory conditions [12].

This study may provide important information concerning the safe traits of *L. plantarum* and *L. paracasei* strains isolated from traditional Sardinian fermented foods. Despite some limitations, including the restricted selection of antibiotics and primers used in this screening, the results revealed a preliminary distinct, species-specific architectures: *L. plantarum* displayed mainly chromosomally encoded and intrinsic resistances, while *L. paracasei* carried several plasmid-associated but non-conjugative determinants. These structural differences likely reflect the ecological and technological contexts of isolation meat-derived environments for *L. plantarum* and dairy matrices for *L. paracasei.* In these contexts, different antimicrobial exposures and microbial communities may exert distinct selective pressures on resistance maintenance and gene organization [42].

From a One Health perspective, these results suggest the surveillance of technologically relevant and QPS-listed LAB remains crucial, considering their wide use as starter or adjunct cultures and their natural presence in fermented foods of both plant and animal origin [43,44,45]. This is because it is essential to preserve microbial biodiversity and product quality; however, it is also necessary to ensure that traditional fermentation ecosystems do not become hidden reservoirs of antibiotic resistance.

## 4. Materials and Methods

### 4.1. Bacterial Isolates and Growth Conditions

A total of 50 LAB strains, including 25 *L. plantarum* and 25 *L. paracasei*, were used in this study. These isolates were originally obtained from traditional Sardinian fermented meat and dairy products by standard culture-based procedures on MRS agar, and species-level identification was performed through phenotypic characterization and confirmed by 16S rRNA gene sequencing, as described in references [46,47].

For the present experiments, strains were grown in MRS (Man Rogosa and Sharpe) medium (Oxoid, Milan, Italy) at 32 °C for 48 h under anaerobic conditions (Gas-Pack; Oxoid, Milan, Italy).

### 4.2. Phenotypic Antibiotics Susceptibility Testing

The antibiotics selected for the analysis of antimicrobial resistance included several agents considered as basic requirements for initial screening [40] and were chosen as representatives of the antimicrobial classes most commonly used in veterinary and food-related settings.

#### 4.2.1. Assessment by Agar Overlay Disc Diffusion

The antibiotic susceptibility screening initially was performed by Agar-overlay Disc Diffusion (ADD) method following the general procedure described by Huys et al., with minor adaptations [28].

Briefly, paper discs impregnated of ampicillin (10 μg), gentamicin (10 μg), streptomycin (10 μg), tetracycline (30 μg) or, vancomycin (30 μg) were placed onto solidified LSM (90% IsoSensitest medium, 10% MRS broth) agar plates inoculated with a bacteria concentration corresponding to 1 McFarland turbidity standard (~3 × 10^8^ CFU/mL) and allowed to solidify at room temperature. After that, the plates were overlaid with 3 mL of MRS soft agar (0.7% agar, *p*/*v*), at 50 °C) and incubated for 24 h at 30 °C under anaerobiosis conditions. The inhibition halo diameters were measured and results (average of three determinations) were expressed in terms of resistance (R), moderate susceptibility (MS), or susceptibility (S), according to interpretative standards detailed in Table 5 [48].

#### 4.2.2. Determination of the Minimum Inhibitory Concentration (MIC)

In addition, antibiotics susceptibility as the lowest concentration of each antibiotic that inhibits the visible growth of bacteria (MIC, Minimum Inhibitory Concentration) were cross validated by MIC Test Strips (Liofilchem, Roseto degli Abruzzi, Italy) according to the manufacturer’s instructions that report same range (0.016–256 µg/mL) for all antibiotics tested in this study. Briefly, bacterial suspensions with a turbidity equivalent to McFarland standard 1 (~3 × 10^8^ CFU/mL) were prepared and inoculated in LSM consisting of 90% IsoSensitest broth (Oxoid, Milan, Italy), 10% MRS broth supplemented with 1.5% agar (Oxoid, Milan, Italy). When the test strip is applied onto an inoculated agar surface, the preformed exponential gradient (across 15 two-fold dilutions like those of a conventional MIC method) of the antimicrobial agent was transferred into the agar matrix.

After 24 h of incubation, a symmetrical inhibition ellipse centered along the strip was formed. The MIC was read directly from the scale in terms of µg/mL, at the point where the edge of the inhibition ellipse intersects with the MIC test strip. MIC values were compared with MIC breakpoints of *Lactobacillus* spp. defined by EFSA 2012 [40].

### 4.3. Detection of Antibiotic Resistance Genes in Genomic and Plasmid DNA

Detection of antibiotic resistance genes was performed by colony PCR, consisting of direct amplification using, as template, a bacterial colony resuspended in 50 µL of sterile water [36]. Plasmid DNA was obtained using Eurogold plasmid Miniprep kit (Euroclone) according to the manufacturer’s instructions. The DNA concentration, integrity and purity were assessed by spectrophotometric measurements using SPECTROstar Nano Microplate Reader (BMG Labtech, Ortenberg, Germany). All the DNA samples were stored at −20 °C.

The presence of resistance genes was determined by PCR amplification using the primers listed in Table 6. The amplification reaction was performed in 25 µL volume, containing 5 µL of the template DNA (approximately 20–50 ng per reaction), 5 mM MgCl_2_, 100 µM DNTPs, and 5 U TAQ DNA polymerase (Invitrogen). The reaction mix was incubated for 5 min at 94 °C and then amplified for 35 cycles consisting in 1 min at 94 °C, 1 min at 50 °C or 55 °C for primers annealing, 1 min at 72 °C followed by 10 min at 72 °C for the final extension. PCR products were separated by 1% agarose gel electrophoresis, stained by SYBR safe (Invitrogen, Thermo Fisher Scientific, Waltham, MA, USA) and visualized with Chemi Doc XRS imaging system (BioRad Laboratories, Milan, Italy). Each PCR run included a no-template control (NTC) to exclude contamination, whereas positive control strains were not used in this assay.

## Figures and Tables

**Table 1:** Phenotypic susceptibility to antibiotics in *L. plantarum* strains.

	Chloranphenicol		Erytromycin		Ampicillin		Tetracycline		Streptomycin		Gentamicin		Vancomycin	
*L. Plantarum* Strains	^a^ ADD	^b^ MIC (mg/L)	^a^ ADD	^b^ MIC (mg/L)	^a^ ADD	^b^ MIC (mg/L)	^a^ ADD	^b^ MIC (mg/L)	^a^ ADD	^b^ MIC (mg/L)	^a^ ADD	^b^ MIC (mg/L)	^a^ ADD	^b^ MIC (mg/L)
PT 0-2	(S)	n.p.	(S)	n.p.	(S)	0.38	(MS)	>256	(S)	96	(R)	>256	(R)	>256
PT 0-4	(S)	n.p.	(S)	n.p.	(S)	0.38	(MS)	>256	(S)	128	(R)	>256	(R)	>256
PT 1-7	(S)	n.p.	(S)	n.p.	(R)	>256	(R)	>256	(R)	96	(R)	>256	(R)	>256
PT 1-8	(S)	n.p.	(S)	n.p.	(R)	>256	(R)	>256	(S)	>256	(S)	>256	(R)	>256
PT 2-3	(S)	n.p.	(S)	n.p.	(MS)	1.5	(R)	12	(MS)	>256	(S)	>256	(R)	>256
PT 3-9	(S)	n.p.	(S)	n.p.	(MS)	1.5	(MS)	16	(R)	>256	(S)	>256	(R)	>256
PT 5-5	(S)	n.p.	(S)	n.p.	(R)	>256	(R)	>256	(R)	>256	(R)	>256	(R)	>256
PT 5-6	(S)	n.p.	(S)	n.p.	(R)	>256	(R)	>256	(R)	>256	(R)	>256	(R)	>256
PT 5-9	(S)	n.p.	(S)	n.p.	(R)	>256	(R)	>256	(R)	>256	(R)	>256	(R)	>256
PT 5-10	(S)	n.p.	(S)	n.p.	(R)	>256	(R)	>256	(R)	>256	(R)	>256	(R)	>256
PT 7-3	(S)	n.p.	(S)	n.p.	(R)	>256	(R)	1.5	(R)	>256	(R)	>256	(R)	>256
PT 9-1	(S)	n.p.	(S)	n.p.	(R)	>256	(R)	>256	(MS)	>256	(R)	>256	(R)	>256
PT 9-2	(S)	n.p.	(S)	n.p.	(R)	>256	(R)	1	(R)	>256	(R)	>256	(R)	>256
PT 9-3	(S)	n.p.	(S)	n.p.	(MS)	0.94	(R)	1.5	(MS)	>256	(S)	>256	(R)	>256
PT 9-8	(S)	n.p.	(S)	n.p.	(MS)	0.94	(R)	>256	(S)	>256	(S)	>256	(R)	>256
PT 9-11	(S)	n.p.	(S)	n.p.	(MS)	0.94	(MS)	0.75	(S)	>256	(S)	>256	(R)	>256
PT 13-3	(S)	n.p.	(S)	n.p.	(MS)	0.38	(MS)	>256	(S)	>256	(S)	>256	(R)	>256
PT 18-1	(S)	n.p.	(S)	n.p.	(MS)	0.38	(R)	>256	(MS)	>256	(R)	>256	(R)	>256
PT 18-2	(S)	n.p.	(S)	n.p.	(MS)	0.38	(R)	>256	(MS)	>256	(R)	>256	(R)	>256
PT 18-3	(S)	n.p.	(S)	n.p.	(MS)	0.38	(R)	>256	(MS)	>256	(R)	>256	(R)	>256
PT 18-6	(S)	n.p.	(S)	n.p.	(MS)	0.125	(MS)	1.5	(S)	>256	(S)	>256	(R)	>256
PT 18-8	(S)	n.p.	(S)	n.p.	(S)	0.38	(MS)	>256	(MS)	>256	(S)	>256	(R)	>256
PT 18-9	(S)	n.p.	(S)	n.p.	(R)	>256	(R)	8	(MS)	128	(R)	>256	(R)	>256
PT 23-1	(S)	n.p.	(S)	n.p.	(MS)	0.5	(R)	>256	(R)	>256	(S)	>256	(R)	>256
PT 23-2	(S)	n.p.	(S)	n.p.	(MS)	0.38	(MS)	4	(S)	>256	(S)	>256	(R)	>256

^a^, Disc Diffusion Method, based on standards mentioned in Section 4, strains were characterized as either susceptible (S), moderately susceptible (MS), or resistant (R) to each antibiotic tested; ^b^, Minimum inhibitory concentration (MIC); n.p., not performed; MIC > 256 µg/mL means a value exceeding the upper limit of the assay.

**Table 2:** Phenotypic susceptibility to antibiotics in *L. paracasei* strains.

	Chloranphenicol		Erytromycin		Ampicillin		Tetracycline		Streptomycin		Gentamicin		Vancomicin	
*L. paracasei* Strains	^a^ ADD	^b^ MIC (mg/L)	^a^ ADD	^b^ MIC (mg/L)	^a^ ADD	^b^ MIC (mg/L)	^a^ ADD	^b^ MIC (mg/L)	^a^ ADD	^b^ MIC (mg/L)	^a^ ADD	^b^ MIC (mg/L)	^a^ ADD	^b^ MIC (mg/L)
18AC1	(S)	n.p.	(S)	n.p.	(S)	>256	(S)	>256	(R)	>256	(R)	>256	(R)	>256
19AC2	(S)	n.p.	(S)	n.p.	(R)	2	(S)	0.25	(R)	>256	(R)	>256	(R)	>256
20AC1	(S)	n.p.	(S)	n.p.	(R)	1.5	(S)	0.19	(R)	>256	(R)	>256	(R)	>256
22AC2	(S)	n.p.	(S)	n.p.	(R)	>256	(S)	>256	(R)	>256	(R)	>256	(R)	>256
23AC3	(S)	n.p.	(S)	n.p.	(R)	1.5	(S)	0.25	(R)	>256	(R)	>256	(R)	>256
25AC3	(S)	n.p.	(S)	n.p.	(R)	0.75	(S)	0.38	(R)	>256	(R)	>256	(R)	>256
26AC3	(S)	n.p.	(S)	n.p.	(MS)	>256	(S)	>256	(R)	>256	(R)	>256	(R)	>256
29AC3	(S)	n.p.	(S)	n.p.	(R)	2	(S)	0.25	(R)	>256	(R)	>256	(R)	>256
38BC1	(S)	n.p.	(S)	n.p.	(R)	>256	(S)	>256	(R)	>256	(R)	>256	(R)	>256
39BC1	(S)	n.p.	(S)	n.p.	(R)	2	(S)	0.5	(R)	>256	(R)	>256	(R)	>256
32AC4	(S)	n.p.	(S)	n.p.	(R)	1.5	(S)	0.25	(R)	>256	(R)	>256	(R)	>256
7BC3	(S)	n.p.	(S)	n.p.	(R)	2	(S)	0.5	(R)	>256	(R)	>256	(R)	>256
34AC3	(S)	n.p.	(S)	n.p.	(R)	1.5	(S)	0.25	(R)	>256	(R)	>256	(R)	>256
35AC3	(S)	n.p.	(S)	n.p.	(R)	0.38	(S)	0.25	(R)	>256	(R)	>256	(R)	>256
36AC3	(S)	n.p.	(S)	n.p.	(R)	3	(S)	0.25	(R)	>256	(R)	>256	(R)	>256
2AM1	(S)	n.p.	(S)	n.p.	(R)	2	(S)	0.5	(R)	>256	(R)	>256	(R)	>256
5AM1	(S)	n.p.	(S)	n.p.	(R)	1.5	(S)	0.19	(R)	>256	(R)	>256	(R)	>256
3AM1	(S)	n.p.	(S)	n.p.	(R)	1.5	(S)	0.38	(R)	>256	(R)	>256	(R)	>256
6ACu2	(S)	n.p.	(S)	n.p.	(R)	3	(S)	0.25	(R)	>256	(R)	>256	(R)	>256
7ACu2	(S)	n.p.	(S)	n.p.	(R)	>256	(S)	0.5	(R)	>256	(R)	>256	(R)	>256
9BC3	(S)	n.p.	(S)	n.p.	(MS)	>256	(MS)	3	(R)	>256	(R)	>256	(R)	>256
10AM3	(S)	n.p.	(S)	n.p.	(R)	2	(S)	0.25	(R)	>256	(R)	>256	(R)	>256
11AM3	(S)	n.p.	(S)	n.p.	(R)	1.5	(S)	0.19	(R)	>256	(R)	>256	(R)	>256
12AM3	(S)	n.p.	(S)	n.p.	(R)	>256	(S)	1	(R)	>256	(R)	>256	(R)	>256
13AM3	(S)	n.p.	(S)	n.p.	(R)	>256	(S)	0.19	(R)	>256	(R)	>256	(R)	>256

^a^, Disc Diffusion Method; ^b^, based on standards mentioned in Section 4, strains were characterized as either susceptible (S), moderately susceptible (MS), or resistant (R) to each antibiotic tested; ^c^, Minimum inhibitory concentration (MIC) n.p., not performed; MIC > 256 µg/mL means a value exceeding the upper limit of the assay.

**Table 3:** Detection and localization of antibiotics resistance genes in *L. plantarum*.

*L. plantarum* Strains	Genomics	Plasmids
PT 0-2	*tet(W)*, *vanX*	*aac(6′)-Ie–aph(2″)-Ia*, *strA*
PT 0-4	*tet(W)*, *vanX*	*aac(6′)-Ie–aph(2″)-Ia*, *strA*
PT 1-7	*tet(W)*, *vanX*	*strA*
PT 1-8	*tet(W)*, *vanX*	*-*
PT 2-3	*tet(W)*	*aac(6′)-Ie–aph(2″)-Ia*, *strA*
PT 3-9	*tet(W)*, *vanX*	*blaZ*, *aac(6′)-Ie–aph(2″)-Ia*, *strA*
PT 5-5	*tet(W)*, *vanX*	*aac(6′)-Ie–aph(2″)-Ia*, *strA*
PT 5-6	*tet(W)*	*aac(6′)-Ie–aph(2″)-Ia*, *strA*
PT 5-9	*tet(W)*	*blaZ*, *strA*
PT 5-10	*tet(W)*	*blaZ*, *strA*
PT 7-3	*tet(W)*, *vanX*	*blaZ*, *strA*
PT 9-1	*aac(6′)-Ie–aph(2″)-Ia*, *tet(W)*, *vanX*	*strA*
PT 9-2	*tet(W)*	*blaZ*, *aac(6′)-Ie–aph(2″)-Ia*, *strA*, *vanX*
PT 9-3	*tet(W)*	*-*
PT 9-8	*tet(W)*	*strA*
PT 9-11	*tet(W)*	*strA*
PT 13-3	*tet(W)*	*strA*
PT 18-1	*tet(W)*, *vanX*	*aac(6′)-Ie–aph(2″)-Ia*, *strA*
PT 18-2	*tet(W)*, *vanX*	*aac(6′)-Ie–aph(2″)-Ia*, *strA*
PT 18-3	*tet(W)*, *vanX*	*aac(6′)-Ie–aph(2″)-Ia*, *strA*
PT 18-6	*tet(W)*, *vanX*	*blaZ*
PT 18-8	*tet(W)*, *vanX*	*aac(6′)-Ie–aph(2″)-Ia*, *strA*
PT 18-9	*tet(W)*, *vanX*	*aac(6′)-Ie–aph(2″)-Ia*, *strA*
PT 23-1	*tet(W)*, *vanX*	*-*
PT 23-2	*tet(W)*, *vanX*	*-*

**Table 4:** Detection and localization of antibiotics resistance genes in *L. paracasei*.

*L. paracasei* Strains	Genomics	Plasmids
18AC1	-	*aac(6′)-Ie–aph(2″)-Ia*, *strA*
19AC2	*strA*	*-*
20AC1	*strA*	*-*
22AC1	*-*	*strA*
23AC3	*-*	*aac(6′)-Ie–aph(2″)-Ia*, *strA*
23AC3	*vanX*	*blaZ*, *strA*
26AC3	*vanX*	*strA*
29AC3	*strA*	*-*
38BC1	*strA*, *vanX*	*blaZ*, *aac(6′)-Ie–aph(2″)-Ia*
39BC1	*vanX*	*blaZ*, *aac(6′)-Ie–aph(2″)-Ia*, *strA*
32AC4	*vanX*	*aac(6′)-Ie–aph(2″)-Ia*, *strA*
7BC3	*strA*, *vanX*	*-*
34AC3	*strA*	*-*
35AC3	*vanX*	*blaZ*, *strA*
36AC3	*strA*, *vanX*	*-*
2AM1	*vanX*	*blaZ*, *aac(6′)-Ie–aph(2″)-Ia*, *strA*
5AM1	*vanX*	*blaZ*, *aac(6′)-Ie–aph(2″)-Ia*, *strA*
3AM1	*vanX*	*blaZ*, *aac(6′)-Ie–aph(2″)-Ia*, *strA*
6ACu2	*vanX*	*aac(6′)-Ie–aph(2″)-Ia*, *strA*
7ACu2	*vanX*	*blaZ*, *aac(6′)-Ie–aph(2″)-Ia*, *strA*
9BC3	*vanX*	*blaZ*, *strA*
10AM3	*vanX*	*aac(6′)-Ie–aph(2″)-Ia*, *strA*
11AM3	*vanX*	*blaZ*, *aac(6′)-Ie–aph(2″)-Ia*, *strA*
12AM3	*vanX*	*aac(6′)-Ie–aph(2″)-Ia*, *strA*
13AM3	*vanX*	*blaZ*, *strA*

**Table 5:** Antibiotics tested and interpretive standards associated with Agar-overlay Disc Diffusion method (ADD).

Classes	Antibiotics	Inhibition Halo Diameter (mm)		
		R	MS	S
β-lactams	Ampicillin (10 μg)	≤12	13–15	≥16
Tetracyclines	Tetracycline (30 μg)	≤14	15–18	≥19
Aminoglycosides	Streptomycin (10 μg)	≤11	12–14	≥15
Aminoglycosides	Gentamicin (10 μg)	≤12	-	≥13
Glycopeptides	Vancomycin (30 μg)	≤14	15–16	≥17
Phenicols	Chloramphenicol (30 μg)	≤13	14–17	≥18
Macrolides	Erythromycin (15 μg)	≤13	14–17	≥18

R, resistant; MS, moderate susceptibility; S, susceptibility.

**Table 6:** Lists of primers to detect antibiotic resistance genes.

Resistence Gene	Target Antibiotic	Forward Primer	Reverse Primer	T (°C)	bp	Reference
***aac(6′)-Ie–aph(2″)-Ia***	gentamicin	5′CAGAGCCTTGGGAAGATGAAG3′	5′CCTCGTGTAATTCATGTTCTGGC3′	50	348	[49]
***strA***	streptomycin	5′ATCCTTCGGCGCGATTTTG3′	5′GCAGCGCAATGACATTCTTG3′	50	282	[50]
***blaZ***	ampicillin	5′TAGGTTCAGATTGGCCCTTAG3′	5′ACTTCAACACCTGCTGCTTTC3′	50	240	[36]
***tet(W)***	tetracycline	5′GAGAGCCTGCTATATGCCAGC5′	5′GGGCGTATCCACAATGTTAAC3′	55	168	[51]
***vanX***	vancomycin	5′TCGCGGTAGTCCCACCATTCGTT3′	5′AAATCATCGTTGACCTGCGTTAT3′	55	454	[52]

Note: T (°C) is temperature of annealing used for PCR; bp is the expected amplicon size.

## Data Availability

The original contributions presented in this study are included in the article. Further inquiries can be directed to the corresponding author.
